# Various effects of 11,12 EET rescue wound healing in a combined model of diabetes and ischemia

**DOI:** 10.1038/s41598-023-33400-y

**Published:** 2023-04-21

**Authors:** Katharina Sommer, Heike Jakob, Theresa Lettenmeier, Dirk Henrich, Jasmina Sterz, Ingo Marzi, Johannes Frank

**Affiliations:** 1 Department of Trauma, Hand and Reconstructive Surgery, Hospital of the Johann Wolfgang Goethe-University, Frankfurt am Main, Germany; 2 Department of Trauma, Hand and Reconstructive Surgery, Marienhausklinik St. Josef Kohlhof, Neunkirchen, Germany

**Keywords:** Adrenal gland diseases, Experimental models of disease

## Abstract

Chronic non healing wounds in diabetic patients still impose a major problem in modern medicine. Especially additional peripheral vascular disease complicates treatment success in these patients. Thus, we analyzed the effects of 11,12 epoxyeicosatrienoic acid (EET) in a combined model of hyperglycemia and ischemia in mice. Hyperglycemia was induced by Streptozotozin 2 weeks prior to wounding. 3 days before wound creation 2 of the 3 suppling vessels of the moue ear were cautherized for ischemia. Either 11,12 EET or solvent for control was applied. Wound closure as well as TNF-α, TGF-β, SDF-1α, VEGF, CD31, and Ki67 were measured. The wounds closed on day 14.4 ± 0.4 standard deviation (SD). 11,12 EET treatment enhanced healing to 9.8 ± 0.6 SD. TNF-α level was augmented on day 9 compared to control and receded on day 18. TGF-β seemed to be elevated all days observed after 11,12 EET treatment. SDF-1α was enhanced on day 6 and 9 by 11,12 EET, and VEGF on day 6 and 18 as well as CD13 on day 3, 6, and 18. 11,12 EET did not alter Ki67. 11,12 EET are able to rescue deteriorated wound healing in a combined model of hyperglycamia and ischemia by resolution of inflammation, augmentation of neovascularization and increasing expression of TGF-β as well as SDF-1α.

## Introduction

Wound healing in diabetic patients still imposes a major problem in the clinical setting. Especially diabetes type 2 patients with additional peripheral vascular disease that is commonly seen in this population, are prone to develop complications^1^. Thus evaluating novel treatment strategies for diabetic wounds should address both hyperglycemia as well as hypoxia by diminished blood flow to the wound side.

Chronic ulcers are marked by a failure in transition from inflammatory to proliferative phase^2^. Accordingly, chronic wounds show an augmented expression of TNF-α^2^. In contrast to this, a shortage of TNF-α level is found when challenged by biofilms, making them more susceptible to infection^3^. High concentrations of TNF-α reduce granulation tissue formation by suppression of TGF-β function^4–6^. Active TGF-β itself enhances wound healing under ischemic conditions as well as in diabetes^7,8^. It has already been shown, that TGF-β function is diminished under diabetic conditions causing a reduction in cell migration and myofibroblast formation^9^.

Besides the inability of chronic wounds to resolve the inflammatory phase, angiogenesis is diminished mainly by a reduced VEGF signaling^10^. VEGF is the most potent angiogenic factor in wound healing promoting endothelial cell activation and migration^11,12^. This enhanced recruitment of vessel forming endothelial cells can be evaluated by the cell–cell adhesion protein CD31^13^. Wounds in ischemic tissues show an enhanced expression of VEGF^14^. Additional administration of VEGF furthermore boosts angiogenesis and enhances survival of ischemic skin flaps^14^. Furthermore, elevation of VEGF expression in wounds also ameliorates angiogenesis and epithelialization in diabetic animals^15^.

Thirdly, diabetic wounds display a reduction in chemokines like SDF-1α^16,17^. SDF-1α is important in wound healing as it is responsible for attracting progenitor cells to the wound side, that in turn help in healing by secreting growth factor^18,19^. Additionally, SDF-1α seems to directly influence keratinocyte capacity for proliferation positively^20^. This supports wound healing and more precisely expressed reepithelialization, as high blood glucose level in diabetes impairs proliferative rate of keratinocytes at least in vitro^21,22^.

In earlier studies, we have already demonstrated that epoxyeicosatrienoic acids (EETs) ameliorate wound healing under normal circumstaces as well as under hypoxic or hyperglycamic conditions. Cytochrom p450 enzymes synthesize these lipid mediators from arachidonic acid. They can take part in regulation of vascular tone, angiogenesis and inflammation.

Streptozotocin (STZ) creates a hyperglycamic metabolic state in mice. In our earlier studies we already investigated wound healing in after STZ injection because we wanted to investigate the wound healing under hyperglycemic conditions without any long term effects of hyperglycemia on the whole organism.

EETs have been shown to protect against the damaging effects of TNF-α in a model of lung injury reducing organ injury^23^. This making them an anti-inflammatory regulator in tissues^24^. Their known proangiogenic properties are at least partly mediated by upregulation of VEGF^24,25^. Additionally, EETs have already been proven to enhance angiogenesis and they can promote SDF-1α expression^25–28^.

So we have already identified EETs as a potent tool to ameliorate wound healing under hyperglycemic as well as ischemic conditions in our earlier work^25–27,29^. Thus it was obvious to investigate the effect of 11,12 EET in a combined model of diabetes and ischemia better mimicking the “real” clinical conditions in diabetic patients.

## Results

### Wound closure and reepithelialisation process during wound healing

We evaluated effect of 11,12 EET on the ischemic ear wounds of the diabetic mice by measuring day of wound closure and the process of reepithelialisation throughout the wound healing process. Diabetes by i.p. injection of STZ was performed 2 weeks prior to induction of ischemia and ischemia by cautherization was induced 3 days prior to wounding. For the calculation of the reepithelializied area, we measured wound size every second day after wounding of the ear.

**Figure 1:**
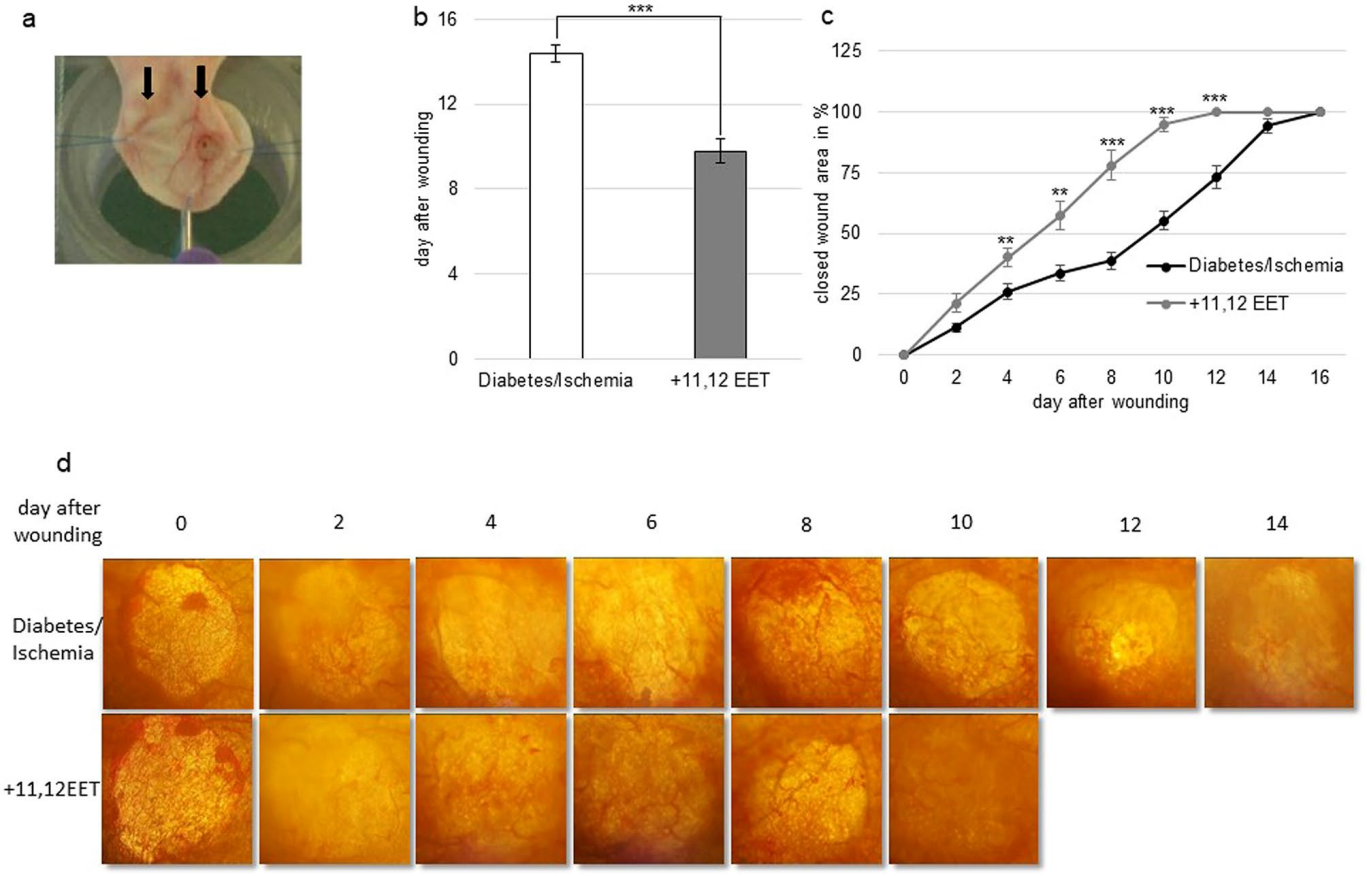
(**a**) Photograph of the wound on the mouse ear. Black arrows indicating the two ligated vessels. (**b**) Day of wound closure. Comparison between non-treated and 11,12 EET treated ischemic wounds in diabetic mice. (**c**) Percentage of closed wound area from day 0–16. Comparison between ischemic wounds of diabetic animals with or without 11,12 EET treatment. (**d**) Representative pictures of ischemic wounds throughout the wound healing process in diabetic mice and after treatment with 11,12 EET (data is shown as mean ± SD; *n* = *10*). **p < 0.01, ***p < 0.001.

After induction of diabetes and ischemia, wounds closed on day 14.4 ± 0.4 standard deviation (SD). The treatment with 11,12 EET enhanced the closure significantly to 9.8 ± 0.6 SD days (Fig. 1b).

The measurement of the closing wound area affirmed this finding as reepithelialization was significantly accelerated after 11,12EET treatment from day 2 to day 12 (Fig. 1c,d).

This illustrates, that local 11,12 EET application ameliorates wound healing under ischemic conditions in diabetic mice.

Furthermore, we analyzed correlation between wound closure and blood glucose level with a group above 400 mg/dl blood glucose level and one below. No correlation between blood glucose level and day of wound closure was found (Table 1).

**Table 1:** Comparison of the day of wound closure in mice exceeding blood glucose level of 400 mg/dl and those below 400 mg/dl.

Blood glucose	Diabetes/Ischemia	+ 11,12 EET
Combined	14.4	9.8
> 400 mg/dl	14.5	10.0
< 400 mg/dl	14.3	9.6

### Evaluation of local inflammation reaction in wounds

Orchestration of local inflammatory reaction is a key step to wound healing. So we evaluated local inflammatory reaction of the ischemic wounds in the diabetic animals by immunohistochemical analysis of TNF-α and TGF-β expression with and without treatment of 11,12 EET. Furthermore, we assessed the expression of the chemokine SDF-1α as one of the main factors for attraction of immune and progenitor cells to the local wound side that are key to regulation of local inflammation.

**Figure 2:**
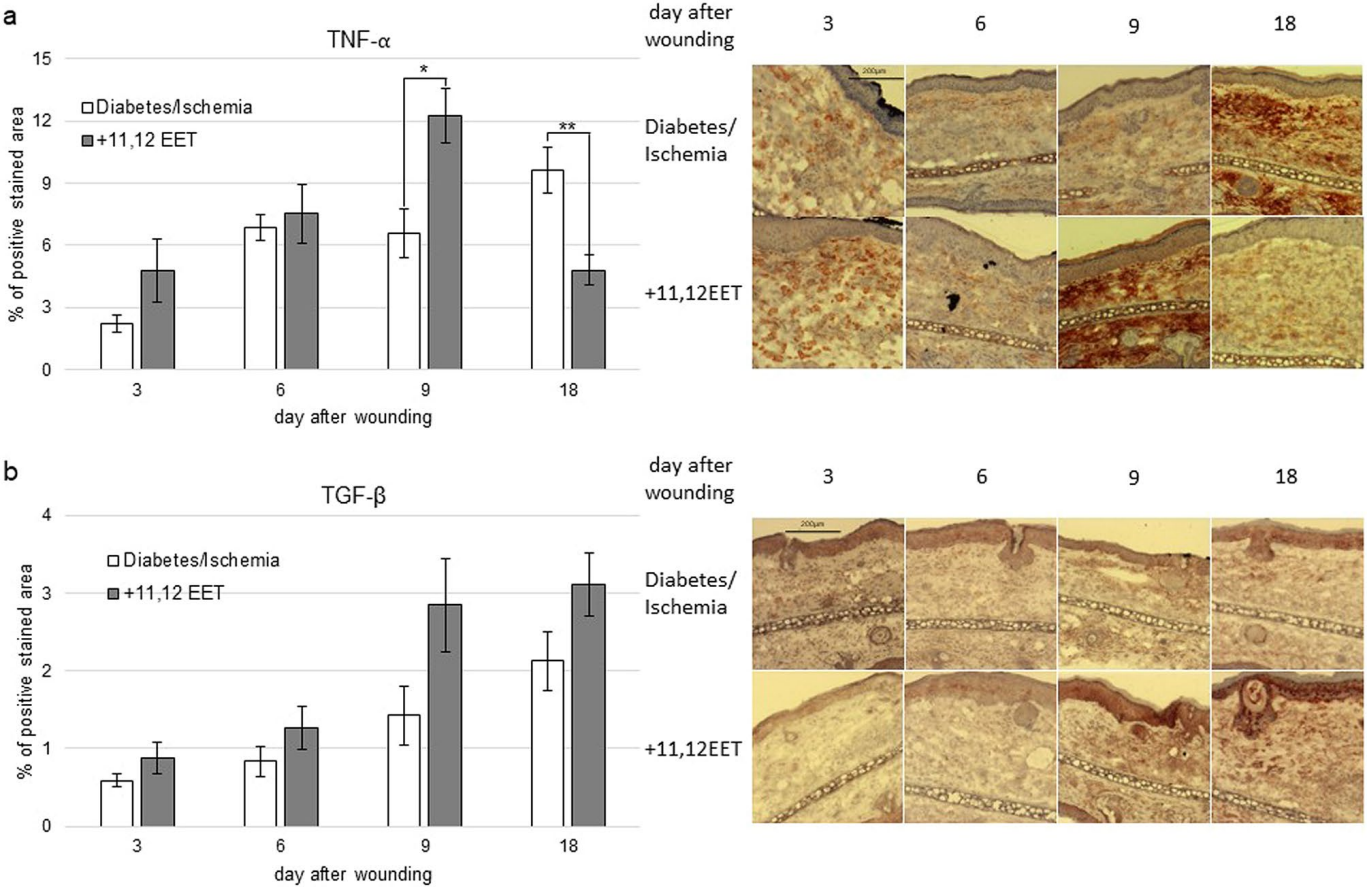
(**a**) Percentage of TNF-α positive area on day 3, 6, 9, and 18 of ischemic wounds of diabetic mice with 11,12 EET treatment and solvent control; on the right, representative pictures of the staining. (**b**) Percentage of TGF-β positive cells on day 3, 6, 9, and 18 of ischemic wounds of diabetic mice with 11,12 EET treatment and solvent control, representative pictures of the staining (data is shown as mean ± SD; *n* = *8* for day 3,6, an 9 *n* = *10* for day 18). *p < 0.05, **p < 0.01.

Expression of TNF-α was not significantly changed by 11,12 EET application on day 3 and 6 though level was slightly higher than in diabetic control on day 3. On day 9, 11,12 EET application enhanced TNF-α expression significantly, whereas on day 16 significantly less TNF-α was found in the 11,12 EET treated wounds (Fig. 2a).

TGF-β expression was not significantly changed by local 11,12 EET application, though an elevation could be noticed on day 9 and 18 after wounding (Fig. 2b).

**Figure 3:**
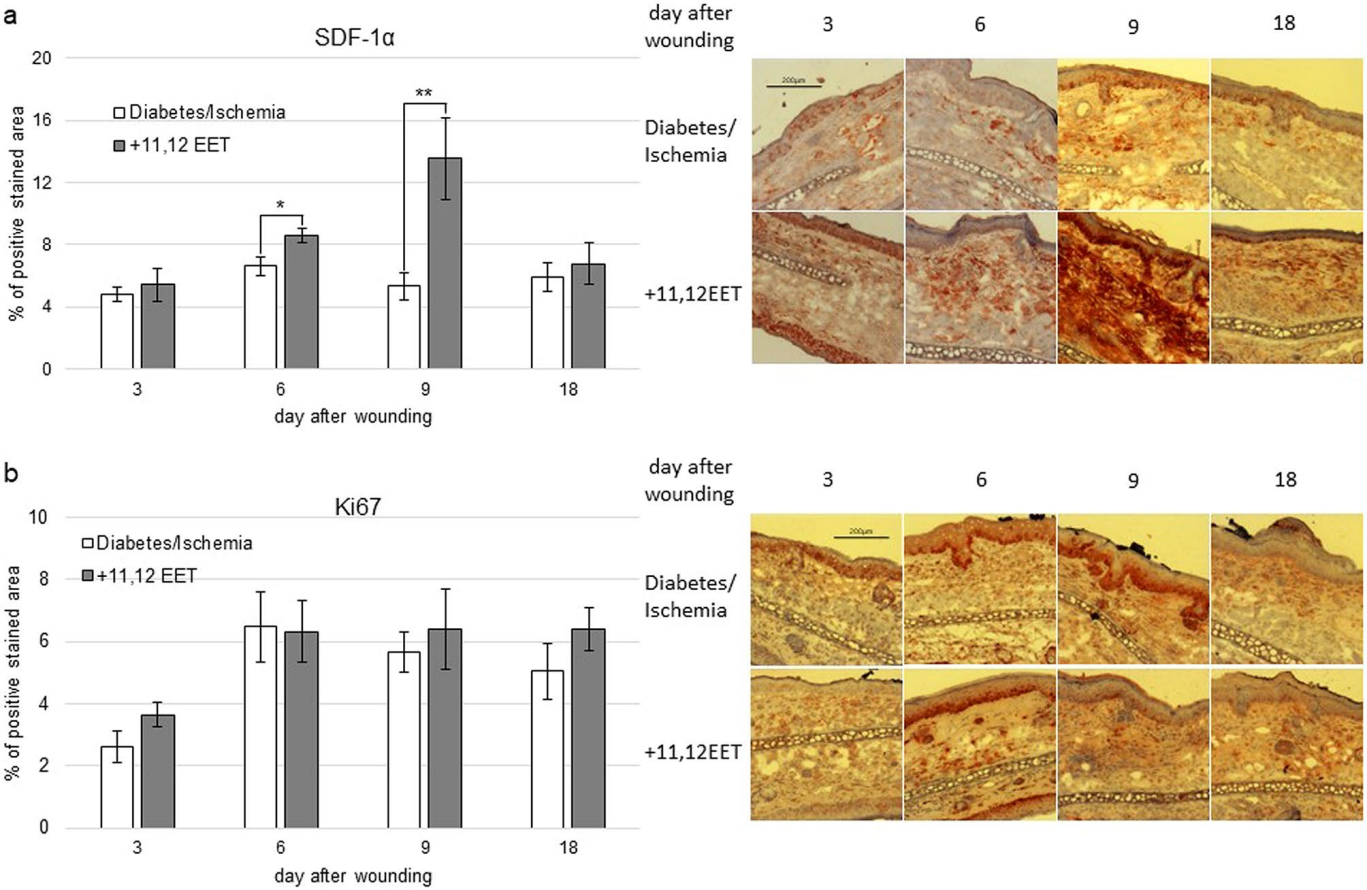
(**a**) Percentage of SDF-1α positive cells on day 3, 6, 9, and 18 of ischemic wounds of diabetic mice with 11,12 EET treatment and solvent control; on the right, representative pictures of the staining (*n* = *12*). (**b**) Percentage of Ki-67 positive area on day 3, 6, 9, and 18 of ischemic wounds of diabetic mice with 11,12 EET treatment and solvent control; on the right, representative pictures of the staining (data is shown as mean ± SD; *n* = *8* for day 3,6, an 9 *n* = *10* for day 18). **p < 0.01.

Concerning SDF-1α expression, 11,12 EET treatment significantly increased the chemokine on day 6 and 9 after wounding (Fig. 3a). On day 3 and 18 SDF-1α level was almost equal in the two groups (Fig. 3a).

### Evaluation of local neovascularisation in wounds

As EETs are known to enhance neovascularisation by elevating VEGF expression, we measured CD31 and VEGF expression in wounds as markers for neovascularisation with and without treatment with 11,12 EETs immunohistochemically.

**Figure 4:**
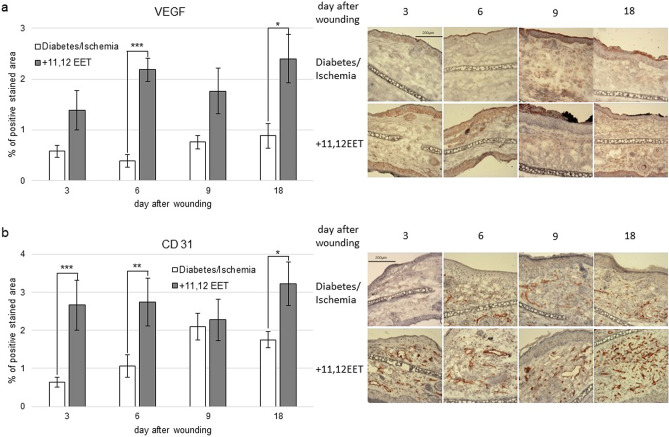
(**a**) Percentage of VEGF positive area on day 3, 6, 9, and 18 of ischemic wounds of diabetic mice with 11,12 EET treatment and solvent control; on the right, representative pictures of the staining. (**b**) Percentage of CD31 positive cells on day 3, 6, 9, and 18 of ischemic wounds of diabetic mice with 11,12 EET treatment and solvent control; on the right, representative pictures of the staining (data is shown as mean ± SD; *n* = *8* for day 3,6, an 9 *n* = *10* for day 18). *p < 0.05, **p < 0.01, ***p < 0.001.

Local 11,12 EET treatment significantly enhanced expression of VEGF on day 6 and 18 after wounding (Fig. 4a). The level in the 11,12 EET group was also higher on the other 2 days investigated, though this finding was not significant (Fig. 4a).

Expression of CD31 was significantly increased on day 3, 6, and 18 in ischemic wounds of diabetic animals after treatment with 11,12 EET compared to control (Fig. 4b). On day 9, CD31 level was equally in both groups.

Summing up, local EET treatment enhances neovascularization as shown by CD31 and VEGF elevation in the early stages of wound healing in diabetic wounds.

### Evaluation of proliferation in wounds

As our wound model hinders wound contraction, the closure is almost entirely achieved by reepithelialization that relies on the proliferating keratinocytes. Therefore we evaluated Ki67 expression as a proliferation marker in the wounds.

We found that 11,12 EET treatment did not significantly alter Ki67 level in the wounds, so there seemed to be a higher level on day 3 and 18 (Fig. 3b).

## Discussion

In this project, we investigated the effect of 11,12 EET on impaired wound healing in in a combined model of hyperglycemia and ischemia. For this purpose, we used the wound model on the ear of the hairless mouse. We induced hyperglycamia by injection of streptozotocin i.p. 2 weeks prior to wounding. Ischemia was created by cautherization of two of the three suppling vessels of the ears.

We found, that local application of 11,12 EET significantly reduced time to wound closure from 14.4 to 9.8 days in hyperglycemic mice under ischemic conditions (Fig. 1b). It also significantly accelerated reepithelialization from day 2 to day 12 (Fig. 1c). These results illustrate that 11,12 EET ameliorate deteriorated wound healing in this combined hyperglycemic and ischemic condition.

In our earlier studies, we already demonstrated that 11,12 EET can rescue wound healing in hyperglycemia as well as ischemia when these two states were considered separately^27,29^. So, the improvement in the combined model was what we already expected. Concerning the time to wound healing in our earlier studies, untreated wounds after induction of diabetes closed around day 13.0, whereas untreated ischemic wound around day 12.8. When we combining these two models, wound healing even worsened with a time to wound healing of 14.4 day, pointing to the fact that the combined model deteriorates wound healing even more than hyperglycemia or ischemia alone.

We further analyzed the molecular mechanisms that helped in improvement of wound healing under hyperglycemic conditions in ischemic wounds by 11,12 EET. Thus, we investigated the expression for TNF-α and TGF-β as well as the chemokine SDF-1α for the inflammatory reaction in the wounds. Concerning TNF-α, there seemed a significant higher expression of the pro-inflammatory cytokine in the wounds on day 9 and significantly less on day 18 (Fig. 2A). This finding is almost analogue to the trend we found in ischemia^27^ hinting to the notion, that EETs might even augment inflammatory reaction in the earlier phases of wound healing but also contribute to the resolution of inflammatory in later stages.

Elevated initial inflammatory reaction might also positively influence wound healing. Initially reduced inflammation accounts at least partly to prolonged wound healing as demonstrated recently in septic mice using the same wound model^30^. The decreased expression of TNF-α on the last day investigated, suggests that EETs are able to restore the necessary transition from inflammatory to proliferative phase in wound healing that chronic diabetic wounds lack^2^.

TGF-β seemed to be elevated on day 9 after wounding, though this finding was not significant (Fig. 2b). TGF-β is an important factor for wound healing. It attracts monocytes, macrophages and fibroblasts to the wound side, thus enhancing angiogenesis and production of extracellular matrix^31^. Furthermore, overexpression of TGF-β was found to be associated with a faster epithelialization of wounds^32^. In diabetes, TGF-β signaling is diminished, thus taking part in the deteriorated wound healing in this state^9^. Consistent with this notion, TGF-β ameliorates wound healing under normal and diabetic conditions^33,34^. So even this slight elevation of TGF-β we found in this study, may take part in the improvement of wound healing in the diabetes ischemia model.

As we already noticed in earlier studies, SDF-1α was elevated in wounds after 11,12 EET treatment^25,27,29^. We could confirm this action by EET in this study as in the treatment group a significant elevation of SDF-1α was found on day 6 and 9 (Fig. 3a). There seemed to be also an augmentation of this chemokine on day 3, though this finding was not significant. SDF-1α is a potent chemokine that attracts progenitor cells to the wound side^18^. Furthermore, it might be able to directly positively influence keratinocyte proliferation positively improving the reepithelialization process^20^. As in diabetes SDF-1α expression is diminished, the augmentation of SDF-1α by 11,12 EET is likely to be another important mechanism that enhances wound healing in the combinational status of ischemia and diabetes^16,17^.

Another important issue in deteriorated healing in chronic wounds is the impaired angiogenesis^35^. Improvement of VEGF expression alone can enhance deteriorated wound healing in diabetes^36^. We also already noted the positive impact of 11,12 EET on neovascularization by elevated levels of VEGF and CD31 after application on wounds in our earlier studies^25–27,29^. EETs have already been noticed to exert this effect as well under other circumstances than wound healing^24^. In this project, we also found an augmentation of VEGF as well as CD31 caused by local 11,12 EET treatment that certainly contributes to the improvement of wound healing in pathologic conditions by 11,12 EET.

It has been demonstrated, that stabilization of hypoxia-inducible factor-1α (HIF-1 α) leads to enhancement of wound healing in diabetes by causing both, the elevated expression of VEGF and SDF-1α^37^. Under normal diabetic conditions, the stability of HIF-1α is decreased. EET are as well able to stabilize HIF-1α in ischemia reperfusion^38^. Thus, the underlying mechanism to enhanced SDF-1 α and VEGF expression by EET might be caused by stabilization of HIF-1 α and further studies will be needed to address this assumption.

Though we found that the 11,12 EET treatment of ischemic ears in diabetic mice did ameliorate wound healing by enhancing neovascularization, SDF-1α expression, resolution of inflammation as marked by TNF-α decrease in later stages of wound healing, and elevation of TGF-β, we did not find a significant augmentation in proliferation that could be monitored by Ki67. And we only noticed a slight increase in the 11,12 EET treated group on day 3 after wounding. Proliferation is an important factor for the reepithelialization process of the wound. So it is surprising, that wounds closed faster though no real elevation in proliferation by measurement of Ki67 was noted. This could be explained partly by the small group size. Another explanation might be that the measurements were taken at the wrong points of time after wounding.

So further studies will be needed to further elucidate the mechanisms that improve wound healing by 11,12 EET e.g. regarding the role of HIF-1α stabilization.

Summing up, 11,12 EET are able to rescue deteriorated wound healing in a combined model of diabetes and ischemia in mice by enhancing neovascularization, resolution of inflammation, and improvement of SDF-1α expression.

## Material and methods

For investigating wound healing under hyperglycamic as well as ischemic conditions we firstly induced diabetes mellitus by STZ followed by cautherization of two of the three suppling vessels to the ear for ischemia. Finally a wound was created on the dorsal side of the mouse ear. We evaluated epithelialization process of the wounds in vivo. Furthermore, neo-vascularization, inflammation, and proliferation was measured by expression of Ki67, TGF-β, TNF-α, VEGF-A, CD31, and SDF-1α on day 3, 6, 9, and 18 after the wounding.

### Animals

Before the start of the animal experiments, ethic approval was obtained by the Regierungspräsidium Darmstadt (Ethic Approval no. V54–19c20/15–F3/17) and the experiments were conducted in conformity with ethic guidelines of German law and also the ARRIVE guidelines.

Male hairless SKH-1 mice (weight 25–35 g; age 8–10 weeks) were accommodated in separate cages at 24 °C with day/night intervals of 12 h/day in airflow regulated rooms and fed a balanced rodent diet with water ad libitum after purchasing the animals from Charles River Laboratories (Sulzfeld, Germany). For anesthesia for surgical procedures as well as wound measurements mice received an intraperitoneal (i.p.) injection of 100 µl solution containing 2.215 mg of ketamine and 0.175 mg of xylazine hydrochloride. At the set time points animals were euthanized by cervical dislocation^25^. No other drugs were used as the pain of the superficial wound on the ear is very low.

### Induction of diabetes

To evaluate the effects of high blood glucose levels in wound healing independent on long time changes, we used STZ to induce hyperglycemia. STZ (Sigma-Aldrich, USA) was injected i.p. solved in 50 mM sodium acetate 1:10 weight adapted in a dose of 0.05 mg/g of body weight over 5 days as described earlier^39–41^. This procedure was done 2 weeks prior to wounding of the ears. Blood glucose level was measured for verification of diabetes by puncturing the tail vein with a blood glucose meter on the day of induction of ischemia, on the day of wounding and every second day afterwards (Accu Chek Sensor & Accu Chek Sensor Comfort Pro- Teststreifen, Roche Diagnostics Deutschland GmbH, Mannheim, Germany). Non-fasting animals with a blood glucose level higher than 250 mg/dl were considered diabetic and were continued to use for induction of ischemia followed by the wound healing experiments. During the experiment, blood glucose was measured every second day for each animal. Furthermore a subgroup analysis of animals with a blood glucose higher than 400 mg/dl and lower than 400 mg/dl was performed. Median blood glucose level of the animals is presented in the table below (Table 2).

**Table 2:** Blood glucose measurements in nonfasted diabetes/ischemia animals with and without treatment with 11,12 EET.

Day after wounding	1	0	2	4	6	8	10	12	14	16	18
Diabetes/ischemia	473 ± 21	418 ± 18	327 ± 27	331 ± 19	342 ± 26	325 ± 22	359 ± 23	371 ± 27	358 ± 29	392 ± 25	459 ± 23
+ 11,12 EET	384 ± 39	319 ± 33	317 ± 36	318 ± 30	316 ± 34	352 ± 47	344 ± 44	331 ± 34	377 ± 40	371 ± 34	389 ± 35

Blood glucose always exceeded 300 mg/dl. (I = day of ischemia).

### Induction of ischemia

For creation of ischemia, 2 weeks after induction of diabetes by STZ, all but one of the three to four neurovascular bundles that supply the mouse ear were cautherized leaving only the most anterior bundle for blood circulation as described previously^42^. This procedure was performed 3 days prior to wound creation on the ears (Fig. 1a).

### Wound creation

After induction of diabetes by STZ and 3 days after creation of ischemia, circular wounds were created on the dorsum of both ears. A full thickness layer of ear skin was dissected down from the underlying cartilage with the help of a circular punch of 2.25 mm diameter as described previously (Fig. 1a)^43^. The wound was covered with a sheet of 2.5% methylcellulose in PBS with or without 11,12 EET according to the treatment group. These methylcellulose sheets were produced by mixing 200 µl of 2.5% carboxymethylcellulose in PBS and adding 200 µl ethanol for control or 160 µl ethanol and 40 µl of 95% 11,12 EET. Then a bioadhesive occlusive dressing was applied to cover the whole mouse ear (Opsite; Smith and Nephew Medical Ltd., Tuttlingen, Germany)^25,43^.

### Wound epithelialization and wound closure measurements

As there is no wound contraction on the mouse ear because of the underlying cartilage, wounds on the dorsal side of the mouse ear allow direct measurement of epithelialization. For this reason, pictures of the wounds were taken on the day of wounding (day 0) and subsequently every second day thereafter. The area of the wounds was calculated on the pictures by computerized measuring of the wound margin reflecting wound epithelialization. For the photographs, the ears of the anesthetized animals were placed outstretched on an acrylic glass platform of a microscope (Carl Zeiss, Oberkochen, Germany) and the image was taken by a camera (DXC-390P, 3CCD color video camera; Sony, Tokyo, Japan). They were then digitalized by a digital converter (ADVC-100; Canopus, Ruppach-Goldhausen, Germany). The wound margin on the images was traced utilizing the ImageJ software for measurement of the wound area (http://rsb.info.nih.gov/ij/download.html). Closed wound area was calculated by using the ratio of the wounded area at each time point divided by the original wound area at day 0. The day of complete epithelialization was defined as day of wound closure. An independent investigator blinded to the group set up conducted the analysis.

### Evaluation of inflammatory reaction, angiogenesis, and proliferation in wounds

After sacrifice of the mice on day 3, 6, 9, and 16 after wounding, the whole ears were embedded in TissueTek (Sakura Finetek Europe, Zoeterwoude, The Netherlands) and stored at − 80 °C. The samples were cut to sections of 6 µm thickness for immunohistochemical staining as described earlier^25^.

The sections were fixed in acetone at − 20 °C for 10 min followed by blocking in 0.1% hydrogen peroxidase for 10 min. Afterwards, staining with primary antibodies directed against VEGF and CD31 (Abcam, Cambridge, UK) for evaluation of angiogenesis, TNF-α, TGF-β, SDF-1α (Abcam, Cambridge, UK) for wound cytokine expression, and Ki67 (Dako, Hamburg, Germany) for proliferation was performed for 1 h at RT or overnight at 4 °C. According to the guidelines of the manufacturer, primary antibodies were detected by suitable secondary antibody with Histofine (Nichirei Biosciences Inc., Tokyo, Japan) or HRP conjugated (Abcam, Cambridge, UK) and stained. Counterstaining was performed by hematoxylin. The stained sections were then examined at 100× magnification analyzing one wound margin in one field for every moue ear (Axio Observer; Carl Zeiss, Oberkochen, Germany). The image was taken with a low light digital camera (AxioCam; Carl Zeiss, Oberkochen, Germany) and analyzed using ImageJ software. The positive area viewed was standardized to the whole area of the image. This analysis was also conducted by an independent investigator.

### Statistical analysis

Data are presented as mean ± SD. It was statistically analyzed using the non-parametric Wilcoxon–Mann–Whitney-U-Test by Bias 10.0 (Epsilon-Verlag, Darmstadt, Germany) 11,12 EET treated group was compared to control at the same points of time. Results with *p* < 0.05 were considered statistically significant. The number of samples examined is indicated by “n”.

## Data Availability

Data cannot be shared publicly. The datasets generated and/or analyzed during the current study are included in the publication; additional data is available upon reasonable request from the corresponding author.

## Abbreviations

EET: Epoxyeicosatrienoic acid
SD: Standard deviation
TNF-α: Tumor necrosis factor alpha
TGF-β: Tumor growth factor-beta
VEGF: Vascular endothelial growth factor
SDF-1α: Stromal cell-derived factor 1α
STZ: Streptozotocin
i.p.: Intraperitoneal
